# Determinants of Bed Net Use in Southeast Nigeria following Mass Distribution of LLINs: Implications for Social Behavior Change Interventions

**DOI:** 10.1371/journal.pone.0139447

**Published:** 2015-10-02

**Authors:** Cheryl L. Russell, Adamu Sallau, Emmanuel Emukah, Patricia M. Graves, Gregory S. Noland, Jeremiah M. Ngondi, Masayo Ozaki, Lawrence Nwankwo, Emmanuel Miri, Deborah A. McFarland, Frank O. Richards, Amy E. Patterson

**Affiliations:** 1 Rollins School of Public Health, Emory University, Atlanta, Georgia, United States of America; 2 The Carter Center, Jos, Nigeria; 3 The Carter Center, Owerri, Nigeria; 4 The Carter Center, Atlanta, Georgia, United States of America; 5 James Cook University, Cairns, Queensland, Australia; 6 RTI International, Dar es Salaam, Tanzania; 7 School of Medicine, University of Alabama, Birmingham, Alabama, United States of America; 8 Ebonyi State Ministry of Health, Abakaliki, Nigeria; 9 Agnes Scott College, Public Health Department, Decatur, Georgia, United States of America; INSERM U1094, University of Limoges School of Medicine, FRANCE

## Abstract

Millions of long-lasting insecticide treated nets (LLINs) have been distributed as part of the global malaria control strategy. LLIN ownership, however, does not necessarily guarantee use. Thus, even in the ideal setting in which universal coverage with LLINs has been achieved, maximal malaria protection will only be achieved if LLINs are used both correctly and consistently. This study investigated the factors associated with net use, independent of net ownership. Data were collected during a household survey conducted in Ebonyi State in southeastern Nigeria in November 2011 following a statewide mass LLIN distribution campaign and, in select locations, a community-based social behavior change (SBC) intervention. Logistic regression analyses, controlling for household bed net ownership, were conducted to examine the association between individual net use and various demographic, environmental, behavioral and social factors. The odds of net use increased among individuals who were exposed to tailored SBC in the context of a home visit (OR = 17.11; 95% CI 4.45–65.79) or who received greater degrees of social support from friends and family (*p*trend < 0.001). Factors associated with decreased odds of net use included: increasing education level (*p*trend = 0.020), increasing malaria knowledge level (*p*trend = 0.022), and reporting any disadvantage of bed nets (OR = 0.39; 95% CI 0.23–0.78). The findings suggest that LLIN use is significantly influenced by social support and exposure to a malaria-related SBC home visit. The malaria community should thus further consider the importance of community outreach, interpersonal communication and social support on adoption of net use behaviors when designing future research and interventions.

## Introduction

Malaria is currently responsible for an estimated 220 million infections and 660,000 deaths, mostly in children less than five years of age, with around 90% of deaths occurring in Africa [1]. Insecticide-treated nets (ITNs) have been shown to reduce the incidence of malaria episodes by 50% in endemic areas [2] and have accordingly become one of the key strategies employed in the global malaria response [3]. Millions of free or highly subsidized ITNs and long-lasting insecticide treated nets (LLINs) have been distributed in the last decade [4–6] resulting in substantial increases in ITN ownership in many malaria-endemic countries [7]. However, multiple studies have revealed that rates of ITN use are often lower than rates of ITN ownership [8, 9]. This presents a significant obstacle to realizing the maximum benefits of ITNs for malaria-related morbidity and mortality since ITNs are maximally protective only when utilized correctly and consistently [10].

Previous studies have explored this apparent “gap” between net ownership and use. Potential determinants of ITN use previously identified include: demographic characteristics [11, 12]; an individual’s knowledge and beliefs related to malaria and bed nets [13–17]; dwelling construction, family size/composition and sleeping arrangements [14, 18, 19]; physical characteristics of bed nets [14, 19, 20]; environmental factors [14, 19, 20]; community and cultural characteristics [21]; and household net density [14, 20]. However, programmatic implications of these findings are not always obvious given that the direction and magnitude of reported associations vary by geographic location, epidemiological setting and method of analysis. This paper examines the determinants of net use through analysis of household survey data collected in southeastern Nigeria and discusses their implications for programmatic interventions designed to increase LLIN use.

Nigeria alone contributes 25% of the African malaria burden [22]. With nearly all of the country’s 160 million people at risk, and an estimated 110 million cases a year, malaria is Nigeria’s most significant public health issue [23]. Since the first national strategic plan for malaria control was introduced in 2006, ITNs, and more recently LLINs, have comprised the central component of the national malaria control efforts [24–26]. At the time of the 2010 Malaria Indicator Survey (MIS) in Nigeria, approximately 42% of households owned at least one ITN and 24% of the people in the total population had slept under any net the previous night, well below the targets of >80% [27]. The analysis of net use among people owning an ITN revealed that only 49% slept under an ITN the previous night, indicating that low net use could not be attributed to low household net ownership alone. Since that time, Nigeria has done much to address the problem of low net ownership, distributing around 56 million nets between 2009 and 2013 in the context of a national campaign [28]. However, there is concern that the lack of accompanying behavior change communication (BCC) to support mass distribution may not lead to significant increases in net use [28].

The analysis presented here contributes to the growing body of work on determinants of net use by assessing the characteristics most strongly associated with net use among an adult population in a malaria-endemic region that has recently completed a statewide mass LLIN distribution campaign, while controlling for the confounding effects of household net density. It also provides insights into the potential mechanisms by which community-based programs like the one introduced in Ebonyi State can motivate increased net use.

## Materials and Methods

The Carter Center, in collaboration with the Nigerian Federal Ministry of Health (FMOH), conducted annual malaria cluster surveys from 2007 to 2011 in the context of a larger study examining the use of community-wide LLIN distributions to interrupt lymphatic filariasis (LF) transmission [29] in rural areas of two local government areas (LGAs) of Ebonyi State: Abakaliki (population = 157,723; 74.5% rural) and Ohaukwu (population = 196,337; 100% rural). This paper presents the results from a secondary analysis of the November 2011 survey data from these two LGAs in order to examine determinants of net use following a mass LLIN distribution campaign and, in select areas, a social behavior change (SBC) intervention described below.

### Survey area

**Fig 1:**
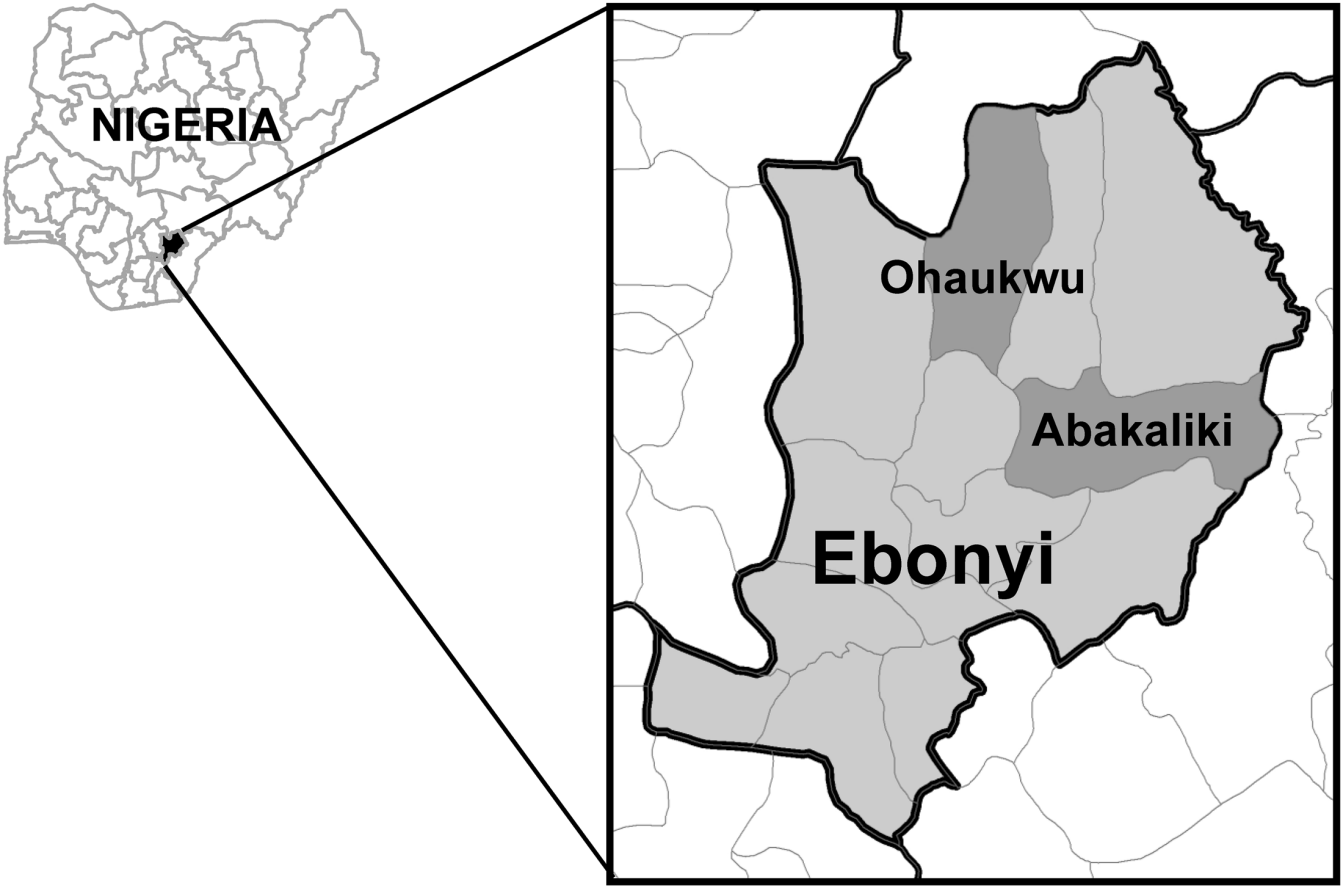
Map of the survey area. Map highlights Ebonyi State in southeast Nigeria, and the Local Government Areas of Ohaukwu and Abakaliki, where the November 2011 survey was conducted.

Ebonyi (Fig 1), a primarily rural state, occupies an area of approximately 5,935 square kilometers and all of its 1.7 million inhabitants are at risk of malaria. *Anopheles gambiae s*.*l*. are the most common vectors for malaria and LF in the area [30], meaning that LLINs can be used to reduce transmission of both diseases. Ebonyi State has two distinct seasons—generally rainy from April to October and dry from November to March. The dominant species of malaria parasite, *Plasmodium falciparum*, is transmitted perennially, although malaria episodes usually peak towards the end of the rainy season [30].

### LLIN distribution

From 2008 to 2010, The Carter Center, in collaboration with the Ebonyi State Ministry of Health, distributed deltamethrin-impregnated PermaNets ^®^ (Vestergaard Frandsen) free of charge in two LGAs of Ebonyi as part of a study to determine the impact of LLIN use on LF transmission [29]: 52,684 LLINs in Ohaukwu targeting vulnerable groups (children under five years and pregnant women), in line with FMOH policy at the time, and 56,680 LLINs in Abakaliki targeting coverage of all household sleeping spaces. Between January and March 2011 a total of 997,492 additional LLINs were distributed in Ebonyi state by the National Malaria Control Program (NMCP) and its partners, including The Carter Center, through a statewide mass LLIN distribution campaign that employed a two-nets-per-household strategy.

### Social behavior change intervention

A community-based SBC intervention to increase the correct and consistent use of LLINs was designed by The Carter Center and implemented through partnership with the Ebonyi State MOH. The intervention was piloted from July to November 2011 within six sentinel villages, three in each study LGA. Community health promoters, selected by their community leaders, carried out the intervention at the household and community levels. SBC intervention activities included: 1) monthly home visits by community health promoters; 2) mobilization of community and religious leaders to support and promote malaria control interventions; and 3) organization of community events including net washing and mending days, workshops to build portable net hanging frames, and malaria-related performances and demonstrations.

During monthly home visits, community health promoters assessed and then addressed each household’s specific barriers to appropriate net use, hanging and care, employing tailored messages and skills-building activities selected from a variety of options. Illustrated flip charts utilized in these visits covered the following: the malaria transmission cycle, the costs associated with malaria and LF infection, the importance of sleeping inside a net every night and in every season, strategies for hanging bed nets over any sleeping space, the correct height for hanging nets, the importance of mending all holes in bed nets, and the appropriate way to wash a bed net. The training materials and job aids emphasized the importance of encouraging social support for net use, based on the belief that social relationships likely play an important role in people’s net use behaviors in Nigeria. The support that people receive from members of their social networks can take a variety of forms, including three that were targeted by this intervention: informational support (information and reminders), emotional support (encouragement) and instrumental support (financial and other tangible resources, direct assistance with a task) [31].

### Household sample selection

**Fig 2:**
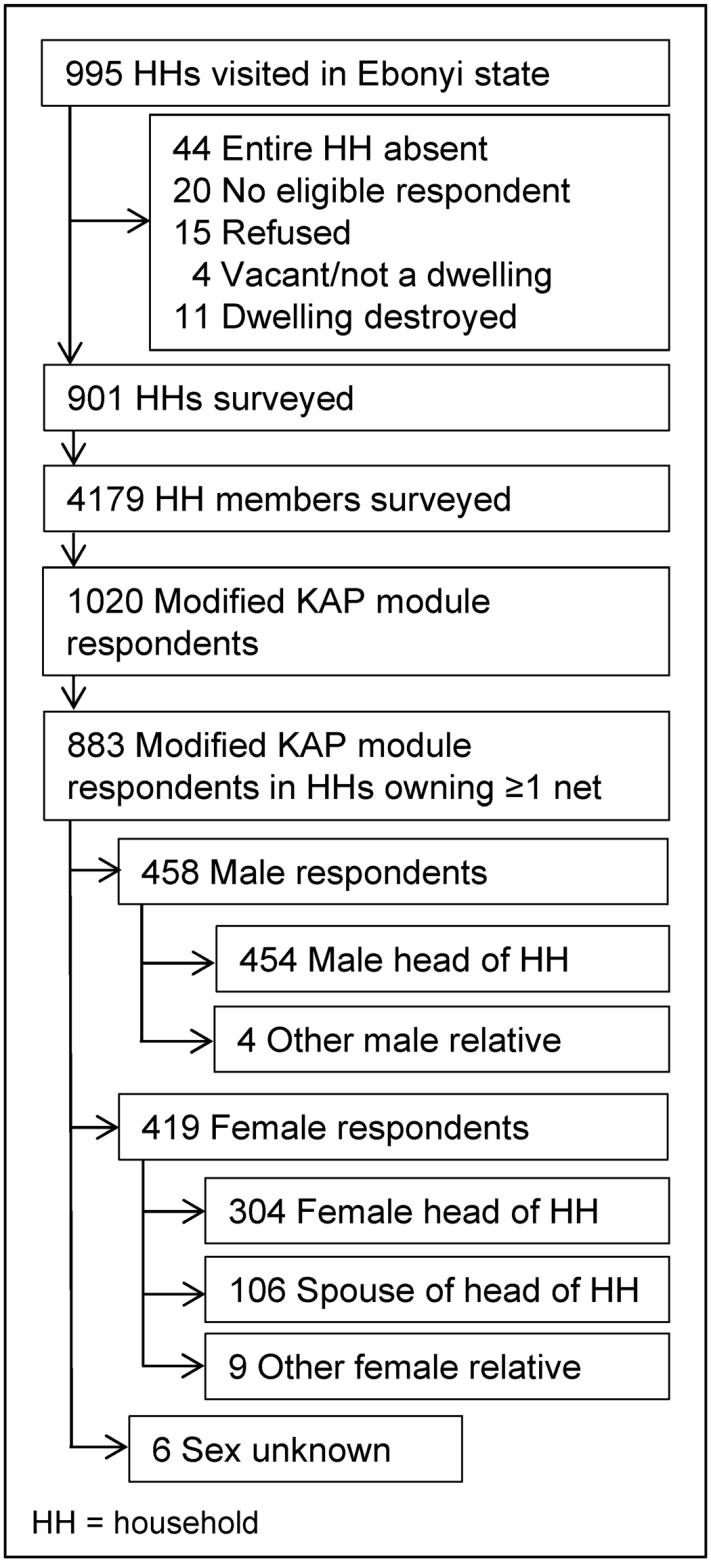
Survey sample. A depiction of the survey sample selection process.

A complete list of census enumeration areas (EAs) was utilized to systematically select 14 rural clusters from both Ohaukwu and Abakaliki LGAs (28 clusters total). An additional 30 clusters were systematically selected from the six sentinel villages where the SBC intervention was conducted to allow for comparative analysis. It was assumed *a priori* that all EAs were of approximately equal size. Twenty-six large EAs were segmented according to the UNICEF Multiple Indicator Cluster Survey (MICS) sampling methodology [32]. All households in the selected EA or segment thereof were included in the survey. The survey team visited 995 households in 58 selected clusters (Fig 2).

### Survey instrument

The survey instrument for the larger survey included standard Malaria Indicator Survey questions on net ownership and use, household and sleeping space characteristics, and demographics. In addition, a customized extended knowledge, attitudes and practices (KAP) module was administered to a subset of adult household members. This module was informed by socio ecological models of health behavior [33], as well as the Health Belief Model [34] and Prochaska’s Stage of Change [35]. In addition to individual factors such as knowledge, attitudes and self-efficacy, the survey measured malaria-related social support [36], social norms, access to information and factors related to both built and natural environment. Data were collected from consenting heads of households and their spouses (in male-headed households) to obtain both male and female perspectives, particularly as women often have great influence over household net use. From one dwelling to the next, trained survey staff alternated between interviewing either the head of household or his spouse (in male-headed households) for the KAP module. In every third household visited, the KAP module was administered to both the head of household and his spouse. In cases where neither the head of household nor his spouse was available, an alternate adult male or female family member was selected. In households in which there were multiple wives, each wife was considered the head of a separate household, following the practice employed during the national LLIN distribution campaign in Nigeria.

### Data entry

Household survey data were collected on paper forms, double entered in Microsoft Access, and subsequently converted to Epi Info 7 to check for consistency and identify any data entry errors using the Data Compare procedures. Data were cleaned in Access and then analyzed using SAS 9.3 (SAS Institute Inc.).

### Statistical analysis

Proportions and means of relevant population characteristics were estimated, adjusting for clustering effects and sampling weights. A net density variable was calculated by dividing the total number of nets in a household by the number of resident household members [14]. This approach, unlike analyses that measure net ownership as the number of household owning a specific number of nets, acknowledges the fact that the number of nets required for a household varies with household size. It thus allows us to separate the effects of insufficient net ownership from other potentially modifiable determinants of net use. Principal components analyses, conducted using the methods of Der and Everitt [37], were utilized to develop several composite variables including: household wealth index [38], net care knowledge, net use skills and self-efficacy, malaria-related social norms, LF knowledge, malaria knowledge, and social support of net use. The social support composite variable included questions pertaining to informational, instrumental and emotional support.

To ensure an appropriate comparison population for individuals who received the SBC intervention and to avoid inadvertently measuring determinants of net ownership (rather than use), only respondents to the extended KAP module residing in Ebonyi State and living in households owning at least one net were included in the analysis of determinants of net use (Fig 2). To assess determinants of net use among respondents, potential explanatory factors with a bivariate association with net use (as measured by self-reported net use last night) at significance *p* < 0.25, according to the procedures of Heeringa *et al*. [39] were selected as candidates for main effects in a multivariable logistic regression model. Regression analysis, assuming absent values were not missing completely at random, was employed to provide more conservative variance estimates. Explanatory factors were removed individually from the model in order of least significance. Independently associated factors at significance *p* < 0.05 (*p*trend < 0.05 for categorical variables with multiple levels) were retained in the final model. To control for the potential confounding effects of differences in net ownership, the continuous net density variable was retained in the final multivariable model. Odds ratios, 95% confidence intervals (CIs) and their corresponding *p*-values were calculated, adjusting for the cluster survey design and sampling strategy.

### Ethical approval

The Ebonyi State Ministry of Health and the Emory University Institutional Review Board approved the comprehensive protocol and use of oral consent for this survey due to high rates of illiteracy in the study area (Emory IRB Protocol #5533). Selected individuals that elected to participate in the survey provided oral consent or assent, which was documented with their survey responses.

## Results

### Household net ownership and use

Table 1 presents weighted estimates of net ownership and use indicators among participating households (n = 901). Overall, 72.2% (95% CI 60.3%-84.1%) of households in the two surveyed LGAs in Ebonyi owned at least one bed net and 60.4% (95% CI 48.4%-72.3%) owned at least two. Households owned an average of 1.52 (95% CI 1.24–1.81) nets per household, with a net density of approximately 0.57 (95% CI 0.52–0.62) nets per person. Greater than 99% of all bed nets owned were LLINs and 80.1% (95% CI 74.7%-85.5%) were used the night prior to the survey. Among households owning at least one net, 74.1% (95% CI 68.6%-79.6%) of individuals reported sleeping under a net the night prior to the survey. Children less than five years of age had the highest proportion of reported net use (79.9%; 95% CI 72.87%-86.97%), while adolescents 15–19 years of age had the lowest (63.6%; 95% CI 53.90%-73.39%).

**Table 1:** Weighted estimates of net ownership and use in 2 LGAs of Ebonyi state, Nigeria, November 2011. HH: Household; LLINs: Long-lasting insecticide treated nets; SD: Standard deviation.

Characteristic	Weighted % or mean (SD)	95% CI	
**Household net ownership (n = 901 HHs)**			
HH with at least one bed net	72.2	60.3	84.1
HH with at least two bed nets	60.4	48.4	72.3
Mean number of nets per HH	1.52 (1.06)	1.24	1.81
Mean HH net density (nets per person)^a^ ^b^	0.57 (0.40)	0.52	0.62
**Net characteristics (n = 1698 nets)**			
Nets that were LLIN (%)	99.8	99.3	100.0
Nets used last night (%)	80.1	74.7	85.5
**Slept under net last night, by age group** ^a^ **(n = 2934 individuals)**			
All ages	74.1	68.6	79.6
Children under age 5	79.9	72.9	87.0
Children age 5–9 years	75.5	68.0	83.0
Children age 10–14 years	69.5	61.0	78.1
Adolescents age 15–19 years	63.6	53.9	73.4
Adults 20–59 years	75.2	69.9	80.6
Persons age ≥60 years	78.5	65.4	91.6

^a^Among households owning ≥1 net.

^b^Does not include baby nets.

### KAP module respondent demographics

Within these 901 households, 1020 persons completed the KAP survey module. Table 2 presents demographic data on the KAP respondents, stratified by SBC villages and non-SBC villages. Nearly half of respondents (48.3%) were female and the vast majority of individuals self-identified as Christians (80.6%) of Igbo ethnicity (97.6%). Other religious affiliations represented included: Traditional (11.0%), Islam (0.7%) and non-religious (4.9%). Farming (including fishing and animal rearing) was reported as the predominant occupation of respondents at 69.1% followed by hand-work/self-employed (8.1%), housewife (6.3%), civil servant (4.6%), trader (4.6%) and student (2.7%). Nearly half of respondents (47.5%) reported having had no formal education, 30.8% attained some primary education, 15.7% attained some secondary education and 4.2% attained some post-secondary education.

**Table 2:** Demographic data of respondents who completed the extended KAP survey module.

	SBC villages (30 clusters)		Non-SBC villages (28 clusters)		Total	
Characteristic	n	%	n	%	n	%
**Total**	525		495		1020	
**Sex**						
Male	284	54.1	234	47.3	518	50.8
Female	236	45.0	257	51.9	493	48.3
Missing	5	0.9	4	0.8	9	0.9
**Religion**						
Christianity	402	76.6	420	84.8	822	80.6
Traditional	81	15.4	31	6.3	112	11.0
Islam	0	0.0	7	1.4	7	0.7
No religion	26	5.0	24	4.8	50	4.9
Missing	16	3.0	13	2.6	29	2.8
**Ethnicity**						
Igbo	515	98.1	481	97.2	996	97.6
Hausa	0	0.0	5	1.0	5	0.5
Fulani	7	1.3	4	0.8	11	1.1
Missing	3	0.6	5	1.0	8	0.8
**Occupation**						
Farmer (fisherman, animal rearer)	395	75.2	310	62.6	705	69.1
Hand-work (self-employed)	34	6.5	49	9.9	83	8.1
Housewife	19	3.6	45	9.1	64	6.3
Civil Servant	23	4.4	24	4.8	47	4.6
Trader (commerce/sales)	24	4.6	23	4.6	47	4.6
Student	8	1.5	20	4.0	28	2.7
Other	14	2.7	20	4.0	34	3.3
Missing	8	1.5	4	0.8	12	1.2
**Education level**						
None	264	50.3	221	44.6	485	47.5
Primary	150	28.6	164	33.1	314	30.8
Secondary	81	15.4	79	16.0	160	15.7
Post-secondary	18	3.4	25	5.1	43	4.2
Missing	12	2.3	6	1.2	18	1.8

### Association between potential determinants and LLIN use

Table 3 presents bivariate logistic regression analysis of associations between explanatory factors and net use among respondents residing in households owning at least one LLIN (n = 883 individuals). At the 95% significance level, net use is associated with several factors including: receipt of a malaria-related home visit (OR = 16.55; 95% CI 6.02–45.50), exposure to the aforementioned SBC intervention (OR = 4.43; 95% CI 2.64–7.44), female gender (OR = 1.77; 95% CI 1.16–2.71), increasing net hanging skills and self-efficacy (*p*trend = 0.04), increasing social support (*p*trend = 0.03) and decreasing malaria knowledge (*p*trend = 0.008).

**Table 3:** Univariable logistic regression analysis between individual net use and explanatory factors among survey respondents living in households owning at least one net, Ebonyi state, Nigeria. CI: confidence interval; SE: standard error; Net density: number of nets per household member; SBC: social behavior change intervention.

Factors	Factor levels	Total	Net used last night		OR	95% CI		*p*-value	*p*-value test for trend
		(n)	%	SE					
**Net density (nets per person)**	<0.05	378	71.11	4.41	1.00	-	-	-	0.11
	0.5–0.9	366	80.27	3.75	1.65	1.03	2.66	0.04	
	≥1.0	133	71.90	8.18	1.04	0.42	2.57	0.93	
**Sex**	Male	458	69.64	4.82	1.00	-	-	-	-
	Female	419	80.24	2.73	1.77	1.16	2.71	0.008	
**Education level**	None	414	81.75	3.69	1.00	-	-	-	0.11
	Primary	284	69.78	5.46	0.52	0.24	1.09	0.08	
	≥ Secondary	171	69.15	6.18	0.50	0.26	0.95	0.04	
**SBC intervention**	No	383	74.70	3.25	1.00	-	-	-	-
	Yes	500	92.90	1.33	4.43	2.64	7.44	<0.0001	
**SBC home visit**	No	563	76.47	3.46	1.00	-	-	-	
	Yes	250	98.18	0.86	16.55	6.02	45.5	<0.0001	
**Malaria knowledge**	Low	310	81.63	3.35	1.00	-	-	-	0.008
	Moderate	220	63.32	6.26	0.39	0.21	0.71	0.002	
	High	323	77.04	3.94	0.76	0.43	1.34	0.34	
**Net hanging skills and self-efficacy**	Low	162	74.62	7.17	1.00	-	-	-	0.04
	Moderate	77	89.11	4.42	2.78	0.86	8.99	0.09	
	High	630	72.35	3.71	0.89	0.42	1.9	0.76	
**Social support**	Low	114	63.88	5.88	1.00	-	-	-	0.03
	Moderate	294	82.90	3.72	2.74	1.28	5.86	0.009	
	High	466	74.82	4.44	1.68	0.89	3.17	0.11	
**Reported any disadvantage of nets**	No	703	78.10	3.35	1.00	-	-	-	-
	Yes	180	65.44	6.62	0.53	0.27	1.04	0.06	
**It is safe to hang a net where food is stored**	Agree	227	73.71	6.19	1.00	-	-	-	0.24
	Neutral	325	79.80	3.88	1.41	0.77	2.59	0.25	
	Disagree	327	70.89	4.01	0.87	0.43	1.76	0.42	

Factors with significance between *p* = 0.05 and *p* = 0.25 utilized in the multivariable model included: net density (*p*trend = 0.112), education level (*p*trend = 0.106), describing any disadvantages of bed nets (*p* = 0.064) and opinion on whether it is safe to hang a net where food is stored (*p*trend = 0.236). Several additional factors were determined to be insignificant in the bivariate analysis and were thus excluded from the multivariable analysis including: age; wealth index; occupation; knowledge of appropriate net use, care and hanging; and net-related stereotypes, rumors and perceived social norms.

The multivariable logistic regression analysis results are presented in Table 4. Controlling for household net density, increased odds of net use among respondents in households owning at least one net is associated with two explanatory factors: receiving a malaria related SBC home visit recently (OR = 17.11; 95% CI 4.45–65.79) and increasing social support score (*p*trend < 0.001), where individuals with moderate and high social support are, respectively, OR = 4.01 (95% CI 1.97–8.16) and 2.22 (95% CI 1.34–3.70) times as likely to sleep under an LLIN as individuals with low social support. Additionally, two factors are associated with decreased odds of net use including: reporting any disadvantage of mosquito nets (OR = 0.39; 95% CI 0.23–0.78) and increasing education level (*p*trend = 0.020), where individuals with primary and secondary or greater education levels were, respectively, 0.43 (95% CI 0.19–0.95) and 0.42 (0.23–0.78) times as likely to use an LLIN as individuals with no education. Level of malaria-related knowledge was also significantly associated with net use (*p*trend = 0.022). Individuals with a moderate level of malaria knowledge were less likely (OR = 0.40; 95% CI 0.19–0.95) to sleep under an LLIN than individuals with low malarial knowledge, however, individuals with high levels of malaria knowledge were similarly likely to sleep under an LLIN (OR = 0.88; 95% CI 0.39–2.02) as those with low knowledge.

**Table 4:** Multivariable logistic regression analysis of association between individual net use and explanatory factors, controlling for net density, among respondents living in households owning at least one net, Ebonyi state, Nigeria. SBC: social behavior change intervention.

Factors	Factor levels	OR	95% CI		*p-value*	*p*-value test for trend
**SBC home visit**	No	1.00	-	-	-	-
	Yes	17.11	4.45	65.79	<0.0001	
**Social support score**	Low	1.00	-	-	-	<0.001
	Moderate	4.01	1.97	8.16	0.0001	
	High	2.22	1.34	3.70	0.002	
**Reporting any disadvantage of nets**	No	1.00	-	-	-	-
	Yes	0.39	0.23	0.78	0.003	
**Education level**	None	1.00	-	-	-	0.020
	Primary	0.43	0.19	0.95	0.036	
	≥ Secondary	0.42	0.23	0.78	0.006	
**Malaria knowledge score**	Low	1.00	-	-	-	0.022
	Moderate	0.40	0.19	0.85	0.017	
	High	0.88	0.39	2.02	0.77	

## Discussion

This study investigated the factors associated with net use among male and female heads of household, and wives of male heads of household, living in two LGAs in Ebonyi, Nigeria following the completion of a mass LLIN distribution campaign and a pilot social behavior change intervention. Findings indicate that the level of social support for net use received from friends and family, exposure to a malaria-related home visit (a component of The Carter Center’s SBC intervention), reporting any disadvantage of nets, education level and degree of malaria-related knowledge are significantly associated with LLIN use in this population.

Our study demonstrates the influence of social support on net use behavior. To our knowledge, this is the first time that such a relationship has been explored in regression analyses of determinants of net use. The relationship between social support and overall health and wellbeing has been described in theory [31, 40], and supported by empirical evidence describing its influence on the adoption of other health behaviors that, like LLIN use, require continuous maintenance: smoking cessation [41], weight loss [42], and medical regimen adherence [43]. Although its importance in other contexts should be confirmed, the relationship between net use and social support presented here suggests a new focus for messages and activities employed in future SBC interventions, possibly requiring tailored and interactive strategies, such as home visits, to aid households in developing new norms regarding social support for net use.

The data suggest that tailored social behavior change interventions that include home visits, as described here, or some other form of interpersonal communication, may be more effective than mass communication campaigns focused on increasing malaria knowledge or emphasizing specific behaviors. Self-reported exposure to SBC home visits was the most significantly associated determinant of net use in this study population. Intervention process data (not presented here) indicate that greater than 90% of households in intervention villages received a home visit; however only approximately half of respondents in these villages reported receiving such a visit. While over-reporting of performance by community health promoters is possible, this result may indicate that the individuals who participated in the extended KAP module during the survey were not home at the time of the home visit and were therefore unaware of its occurrence. This information, combined with the lack of observed association between residence in an intervention village and net use, suggests that home visits may have the strongest impact on directly exposed individuals, and that there may be limited diffusion of information between household members. Such findings suggest the importance of including entire households in a home visit. Additional exploration of the social environment in which net use behaviors are adopted and encouraged should be the focus of future studies and should inform behavior change strategies aimed at increasing appropriate and consistent net use in a population once barriers to net ownership have been reduced.

It is not surprising that survey participants who reported LLIN disadvantages were significantly less likely to not use nets. Further investigation of the specific disadvantages most strongly associated with net non-use may highlight areas for improving LLIN design, distributions and communication messages. Less intuitive, perhaps, is the finding that greater malaria-related knowledge is associated with decreased odds of net use in the examined population. Such results, though relatively uncommon, are not unprecedented. The literature regarding the relationship between malaria knowledge and use of bed nets is conflicting, reporting positive [44–46], negative [47–49] and no [50–52] association. It has been suggested that perhaps only specific variants of malaria-related knowledge are associated with net use [44] thus signifying the importance of the content and delivery of knowledge-related survey questions. In this study, variables included in the knowledge score measured a respondent’s possession of medically correct knowledge of malaria causes, prevention and treatment. Although social desirability bias may continue to influence results, a further understanding of this relationship may be elicited by examining answers to individual questions rather than a composite score and adjusting knowledge questions so as to consider whether individuals believe the facts they report or possess significant local disease knowledge that runs contrary to medically correct knowledge.

Our results also suggest that individuals with higher education are less likely to sleep under a net than less educated individuals. Given that we did not find wealth or occupation to be significantly associated with net use in our study, the effects of education cannot be explained in terms of potentially greater access by the wealthy to window screens, air conditioning or improved housing, any of which might explain decreased net use. A state-wide survey conducted in neighboring Abia state in 2010 also found that net use was not associated with wealth [53]. Generally the literature on education and net use reports that increasing education level is associated with increased net use [12, 54]. However, a 2012 study examining 2008 Nigerian Demographic and Health Survey data found that lower education level was significantly associated with increased net use [55]. The authors of that study hypothesized that the perceived malaria risk may be higher among poorer, less educated populations. Further examination of the relationship between education level and lack of, or improper use of, bed nets may shed light on the underlying factors involved in this association.

The study had some limitations when it comes to describing the processes by which the Social and Behavior Change intervention created behavior change. We were not able to accurately measure the dose of exposure to the behavior change intervention received by each study participant or assess the pathways by which information about net use spread between community members. Though Community Health Promoters collected information about the number of visits to each household, the net ownership and use in the household, in addition to the specific information provided and activities conducted during the visit, we were not able to link households included in the survey to their specific data in the Community Health Promoter registers as a way to cross-validate the participants responses and retention of information. As is the case in all studies of net use, the data are vulnerable to social desirability bias, and those who did receive home visits might have felt more pressure to report net use the previous night. The study was powered to detect associations between exposure to the intervention and net use, but was not sufficiently powered to assess effects of the intervention on malaria infection.

## Conclusion

We present here a novel description of the positive influence of social support (*p*trend <0.001) and SBC home visits (OR = 17.1, 95% CI 4.45–65.79) on net use. These results suggest that interventions focusing on mass media campaigns to increase malaria-related knowledge may not have as positive an effect on net use as tailored interpersonal interventions involving home visits. Future malaria programs should therefore consider shifting from the usual mass communication campaign model to community-based interventions involving household visits that incorporate methods of increasing community outreach and social support for net use among participants in order to increase net use compliance.
